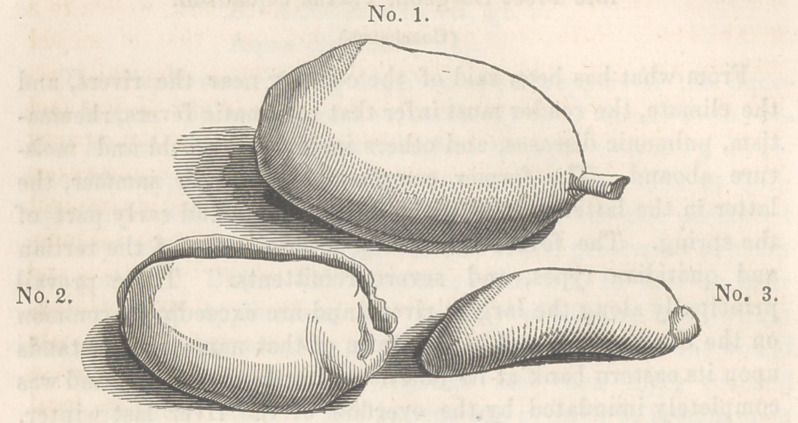# Remarks on California

**Published:** 1851-02

**Authors:** G. R. B. Horner

**Affiliations:** Late Fleet Surgeon, Pacific Squadron


*Remarks on California.* By G. R. B. HORNER, M. D., U. S. N., late Fleet Surgeon, Pacific Squadron.

(Concluded.)

From what has been said of the country near the rivers, and the climate, the reader must infer that miasmatic fevers, rheumatism, pulmonic diseases, and others incidental to cold and moisture abound. The former complaints prevail in summer, the latter in the latter part of the fall, the winter and early part of the spring. The fevers are mostly intermittents of the tertian and quotidian types, and severe remittents. These prevail principally along the largest rivers, and are exceedingly common on the Sacramento, and in the town of that name which stands upon its eastern bank at its junction with the American, and was completely inundated by the overflow of the river last winter. The banks then sloped backwards; the dykes formed were insufficient to keep off the water—it ran around the back of the town—was eight to ten feet deep in some places, and drowned a number of the inhabitants. In July I saw several of the boats used for transit between their houses still lying upon the ground behind them, and among the majestic oaks which shade the banks and rival in height the noble sycamores, which overhang the river, and the numerous vessels anchored along its margin. Among the multitudes of emigrants in California, the typhoid fever, phthisis pulmonalis, scurvy, diarrhœa and dysentery prevail, but the last is most fatal, and has had numerous victims. When conjoined with scurvy it is very fatal. The most severe cases of dysentery, I met with at San Francisco; and in the post mortem examination of one at the city hospital, owned by its able superintendent Dr. Peter Smith, we found the most intense inflammation, with ulceration extending the whole length of the alimentary canal.

Another similar case was under treatment. The nitrate of silver in large doses, even to the amount of 10 and 15 grains, was the principal remedy employed. I understood from him, that he likewise used for the cure of that complaint, and intermittent, the kernels of the Cedron,* a fruit obtained at Panama.

*Some account of this fruit, with its therapeutical uses, will be found in the "Record" of this number.—EDS. EX.

They are an inch and a half long, bi-lobed, brown externally, white within, intensely bitter, and contained in an oval pod.* He

No. 1.

No. 2. No. 3.

gave them scraped, in the dose of 10 or more grains, and thought them equally as efficacious as quinine. The cedron has great celebrity, among the people of the Isthmus, for the cure of snake bites; and he states that they carry it about their persons, to have it in readiness for immediate use when needed. Dr. Smith gave me a number of the kernels and one entire pod, which I brought home for distribution. While crossing the Isthmus I would have been pleased to find the tree which yields this valuable medicine, but I was unable to obtain any accurate description of it, or of the places where it grows. We may, however, be able, after a time, to learn its history, through some of the many travellers passing Panama.

On board the Savannah a number of scorbutic cases occurred while she laid at Saucelito, four miles from St. Francisco. Other cases occurred in the Ohio while there, and several in different vessels of the squadron. Those in the Savannah happened last December and January, and were ascribed not more to the want of vegetable food, than to the constant dampness and chilliness of the air, which induced perfect torpor of the skin, suppressed all perspiration, and induced livid blotches upon it by stagnation of the venous blood. Small dark red specks, like flea-bites, on the lower extremities, swelling of these, and

* No. 1. The pod of the cedron. 2. The two lobes stripped. 3. A side view of one of them.

severe pains in the joints, were other prominent symptoms. Soreness of the gums was another, but in only two instances was it severe. In one of them they became fungous and swollen to a great size. Death occurred only in one case, that of an old marine, who had scurvy joined with dysentery originally acquired during a campaign in Mexico. Not aware of this fact and of his liability to that complaint, I treated him for scurvy after the ordinary manner, allowed him lemonade, apple-water, vegetable diet and bitters, and in this manner probably brought on the dysentery. In the treatment of the scurvy, in conjunction with regimen, the extract of cinchona and gentian, and sulphate of quinine were beneficially used. Warm bathing, frictions of various sorts, poultices, alum, solutions of the sulphate of zinc, and the muriated tincture of iron, were other remedies employed; the last one internally, the others for various local affections of the limbs and mouth. To reduce the swelling of the gums scarification was indispensable. Warm clothing was worn and other comforts allowed the patients when they could be procured. But the supply of fresh provisions was very imperfect, few could get more than dried apples, some wild water cresses and potatoes to eat, and none had the benefit of a hospital of any kind. Our government owned none: private ones were located in the chief towns, but the charges were enormous, accommodations bad, and temptation to desertion so strong that our men could not be trusted out of the ships. I made many fruitless endeavors to get a naval hospital afloat or on shore. Benicia was strongly recommended on account of its superior climate, water, fuel, convenient location, and building materials, and we hope that when labor has become reduced to a reasonable rate, that a hospital will be erected there suitable to the wants of the large naval force we shall have to keep on the coast of California. As for our merchant seamen, and many invalids on shore, they were worse off than our men, who had at least dry bedding and a shelter from the weather. Drugs and physicians were abundant in some parts, but the former were so dear and bad in many places, that patients had to choose between sickness and starvation, and often to prescribe for themselves. Hence, among other instances, I met with a very respectable citizen who had the scurvy, who asked my advice, and on examination was found with his legs covered with slices of raw potatoes held on by bandages. When asked if he thought the former had been of service, he said he thought they had, as they had *become black.* I expressed my doubts on the subject, and recommended he should apply the potatoes internally. In numerous other instances I was called on to prescribe privately, and in several for surgical complaints. Of the latter the most important was that of a young emigrant, who had dislocated his right arm six months previously while on his passage around Cape Horn. He was very anxious to have the bone restored to its place. I made several attempts for this purpose, with and without pullies, but, as he was informed of the risk he ran of having the axillary artery torn, he became afraid of any more attempts being made, and went up to the mines to seek his fortune, crippled as he was.

In coming home I met with two other emigrants who had been injured, one had blown off a thumb in firing a pistol, the other had been knocked down, robbed, and had broken his right thigh bone, for the cure of which he had paid a thousand dollars, and yet had it shortened an inch and a half. In a third case, the patient had had a portion of the bones of the right fore-arm blown off by the accidental discharge of a gun, and had been obliged to suffer amputation on board one of the ships of the squadron, to which he was brought for treatment. In a fourth instance I went two miles ashore to hold a coroner's inquest, on the body of another young emigrant, Thomas S. Davidson, of New Orleans, who had gone out to chop wood and shoot game; but so far as we could ascertain, he had shot himself accidentally when loading one barrel of his gun, while the other was charged. We came to this conclusion from finding a ramrod some feet from the gun, one of the barrels discharged, and the fatal wound extending obliquely upwards through the anterior part of the thorax, from the left to the right side, and in the direction a gun is held when being loaded. His hatchet was found beneath the branches of a low widely-spread horse chesnut which he had chopped in eight different places; and his body lay prostrate on a lawn about twenty yards distant. Convinced from these circumstances that he had come to his death accidentally by his own hands, and that his brother huntsmen, who had their tent pitched in a neighboring valley, were free of all suspicion of being concerned in his untimely end, we had him wrapped in one of their blankets, and buried in a grave dug by our boat's crew on the spot. My worthy companion, Lt. Leroy, and myself then returned to the flag ship, and made an official report of the inquest to Commodore Jones. Many more unhappy incidents, similar to those mentioned, have occurred in the country, and when we take into consideration the great number of our countrymen and others who must suffer from like accidents and from disease, it must seem incumbent on us to provide for them the means of being relieved, and above all to prepare for the indigent, hospitals well built, and furnished with all the necessaries calculated to restore them to health, or add to their comfort as long as they live. For adult males there were hospitals at St. Francisco, Benicia, Sacramento, and Marysville. In the city hospital of the first place, $4 per day were paid by the corporation for every public pauper, and in the marine hospital patients who have not paid the capitation tax of $2, are charged $28 per week. In all other hospitals known to me not less than $5 a day were charged, and in that of Benicia, owned by Dr. Peabody, $10 a day were paid. But in all California I do not know of a single hospital, public or private, where a child or a woman could be, for any price, provided with proper attendance and even ordinary accommodations, and the recent destruction by fire of the city hospital at St. Francisco, will occasion still greater suffering among invalids of every description, but especially to those affected with cholera, which has just made its appearance in California.

## Figures and Tables

**Figure f1:**